# Zinc Deficiency Blunts the Effectiveness of Antidepressants in the Olfactory Bulbectomy Model of Depression in Rats

**DOI:** 10.3390/nu14132746

**Published:** 2022-06-30

**Authors:** Anna Rafało-Ulińska, Bartłomiej Pochwat, Paulina Misztak, Ryszard Bugno, Agata Kryczyk-Poprawa, Włodzimierz Opoka, Bożena Muszyńska, Ewa Poleszak, Gabriel Nowak, Bernadeta Szewczyk

**Affiliations:** 1 Department of Neurobiology, Maj Institute of Pharmacology, Polish Academy of Sciences, 31-343 Krakow, Poland; pochwat@if-pan.krakow.pl (B.P.); nowak@if-pan.krakow.pl (G.N.); szewczyk@if-pan.krakow.pl (B.S.); 2 School of Medicine and Surgery, University of Milano-Bicocca, 20900 Monza, Italy; paulina.misztak@gmail.com; 3 Department of Medicinal Chemistry, Maj Institute of Pharmacology, Polish Academy of Sciences, 31-343 Krakow, Poland; bugno@if-pan.krakow.pl; 4 Department of Inorganic Chemistry, Faculty of Pharmacy, Jagiellonian University Medical College, 30-688 Krakow, Poland; akryczyk@gmail.com (A.K.-P.); mfopoka@cyf-kr.edu.pl (W.O.); 5 Department of Pharmaceutical Botany, Faculty of Pharmacy, Jagiellonian University Medical College, 30-688 Krakow, Poland; bozena.muszynska@uj.edu.pl; 6 Laboratory of Preclinical Testing, Chair and Department of Applied and Social Pharmacy, Medical University of Lublin, 20-093 Lublin, Poland; ewa.poleszak@umlub.pl

**Keywords:** olfactory bulbectomy, zinc deficiency, treatment-resistant depression

## Abstract

Currently used antidepressants do not always provide the desired results, and many patients suffer from treatment-resistant depression. Clinical studies suggest that zinc deficiency (ZnD) may be an important risk factor for depression and might blunt the effect of antidepressants. This study aimed to examine whether ZnD might blunt the effectiveness of antidepressants in the olfactory bulbectomy model (OB) of depression in rats. For this purpose, rats were subjected to the OB model, fed a zinc-deficient diet (3 mg Zn/kg) for 3 weeks, and finally treated with escitalopram (Esc), venlafaxine (Ven) 10 mg/kg, i.p., or combined Esc/Ven (1 mg/kg, i.p.) with zinc (5 mg/kg) for another 3 weeks. Open field (OFT), forced swim (FST), and sucrose intake (SIT) tests were used to evaluate depressive-like behavioral changes. In addition, serum, intracellular, and synaptic Zn concentrations and the level of zinc transporter (ZnT) proteins were analyzed. The OB + ZnD model induced hyperactivity in rats in the OFT, increased immobility time in the FST, and anhedonia in the SIT. Chronic treatment with Esc reduced immobility time in the FST in the OB + ZnD model. Esc/Ven +Zn increased sucrose intake in rats from the OB + ZnD group. The OB + ZnD decreased serum zinc levels and intracellular and synaptic Zn concentration in the prefrontal cortex (PFC) and cerebellum. These changes were normalized by chronic administration of Esc/Ven +Zn. Moreover, OB + ZnD decreased levels of the ZnT1 protein in the PFC and Hp and ZnT3 in Hp. Chronic administration of antidepressants did not alter the levels of ZnT proteins. The OB + ZnD model induces more depressive-like effects than either model alone. Our results show that ZnD may induce drug resistance in rats. Normalizing serum or brain zinc concentration is insufficient to reverse behavioral abnormalities caused by the OB + ZnD model. However, zinc supplementation might improve the effectiveness of antidepressants in reversing particular depression symptoms.

## 1. Introduction

Major depressive disorder (MDD) is a serious medical problem that generates enormous economic and social costs worldwide. It is the second most common disease globally, and the morbidity associated with the disorder has been rising [1,2]. This presents a considerable challenge in terms of effective treatment strategies. The most severely affected depressed patients are those with treatment-resistant depression (TRD) [3]. These patients do not respond to at least two attempts at antidepressant treatment with drugs of different pharmacological classes administered at the appropriate dose for a sufficient period [4,5]. Prevalence studies typically report that more than 50% of depressed patients are resistant to antidepressant drug treatment [6]. A significant advancement in understanding the pathophysiology of depression has been made in the past two decades, primarily due to animal models of depression [7]. One commonly used rodent model of depression is the olfactory bulbectomy model (OB). Removal of the olfactory bulbs in rodents induces endocrine, immune, and neurotransmitter systems and behavioral alterations that resemble the symptoms observed in patients with major depression. The olfactory bulbs, hippocampus, and amygdala are part of the brain’s limbic system responsible for emotions and memory. Behavioral alterations observed after OB are thought to result from dysfunctions or/and compensatory mechanisms of the cortical–hippocampal–amygdala pathway in depressed patients [8]. OB induces hyperactivity, social behavior change, learning and memory deficits, and changes in taste-aversion behavior [9,10]. However, chronic treatment with antidepressants restores OB-induced behavioral, endocrine, immune, and neurotransmitter changes [9,10].

In recent years, clinical and preclinical studies have shown that zinc plays a significant role in the pathophysiology of depression. Among others, it was found that reduced dietary zinc intake may lead to MDD [11,12]. For instance, the level of decline in serum zinc levels has been known to determine the severity of MDD symptoms. Interestingly, serum Zn levels are normalized with antidepressant treatment, albeit not in individuals with treatment-resistant depression [13,14]. Also, in preclinical studies, zinc deficiency has been known to induce depressive-like behavior in adult rodents. Animals fed a zinc-deficient diet exhibited increased immobility time in the forced swim test (FST) and the tail suspension test (TST) [15,16]. Increased immobility time in the FST in ZnD rats was normalized by two weeks of treatment with the serotonin selective reuptake inhibitor (SSRI) fluoxetine [17].

Depression is a heterogeneous disease characterized by different human symptoms, impossible to model in rodents. As was mentioned above, available animal models only model specific symptoms of depression like anhedonia in chronic mild stress, chronic unpredictable stress, or restraint stress [18,19,20]. On the other hand, the OB model is considered a model of agitated depression [21]. In animals subjected to the zinc deficiency model, anhedonia and social impairments were observed. To bring animal models closer to the situation observed in human depression, we simultaneously subjected rats to a combined zinc deficiency and OB model. The expectation was that we would be able to model more symptoms of depression all at once, increasing the predictive validity of the rodent models and/or making them more valuable in studying the etiology of TRD. To explore this hypothesis, we evaluated the behavioral changes induced by OB + ZnD in rats using common behavioral tests such as the open field test (OFT), forced swim test (FST), and sucrose intake test (SIT).

We also studied the effects of chronic treatment with two antidepressant drugs (escitalopram and venlafaxine) commonly used in the treatment of TRD to ameliorate the depressive-like behaviors induced by the OB + ZnD model and the possible mechanisms underlying the accompanying behavioral deficits.

## 2. Materials and Methods

### 2.1. Animals and Housing

Experiments were carried out using male Sprague Dawley rats (220–250 g; 7 weeks old). Rats were maintained on a normal day–night cycle (light phase 7:00–19:00), temperature (22–24 °C), and 40–50% humidity with ad libitum access to food and water. The animals were housed 4 per cage (except rats used in the sucrose intake test, which were housed individually).

### 2.2. Olfactory Bulbectomy—Surgical Procedure

Rats underwent bilateral olfactory bulbectomy under anesthesia (ketamine (100 mg/kg)/xylazine (10 mg/kg)) after one week in the laboratory. Rats were treated with meloxicam (0.05 mg/kg, s.c.) for pain and inflammation one hour after OB and two days after OB. Olfactory bulbs were suctioned through burr holes drilled in the coordinates 7 mm anterior to the bregma and 2 mm (on either side) from the middle line. Burr holes were repaired with a hemostatic sponge (Ferrosan, Poland), and the skin was sutured. Control rats underwent a similar procedure. However, the bulbs were left intact, and animals were allowed to recuperate for one week after surgery. During this time, animals were handled daily to reduce the development of aggressive behavior [22].

### 2.3. Zinc Deficiency Diet

After olfactory bulbectomy, the rats were fed a standard diet with 35 mg Zn/kg for one week. On day 8 after surgery, a zinc-deficient diet was started. The animals were divided into two groups: rats fed a zinc adequate diet (ZnA) of 50 mg Zn/kg or a zinc-deficient diet (ZnD) of 3 mg Zn/kg for 6 weeks. All of the diets were purchased from Altromin GmbH (Lage, Germany).

### 2.4. Drug Administration

Antidepressant therapy was initiated after 3 weeks of feeding. Escitalopram (Esc, 10 mg/kg) or venlafaxine (Ven, 10 mg/kg) or a combination of antidepressants (escitalopram or venlafaxine inactive dose 1 mg/kg; i.p.) and Zn (5 mg/kg; i.p.) was administered (intraperitoneally; i.p.) chronically once daily for 21 days. The remaining rats received saline (0.9% sodium chloride i.p.).

**Scheme 1:**
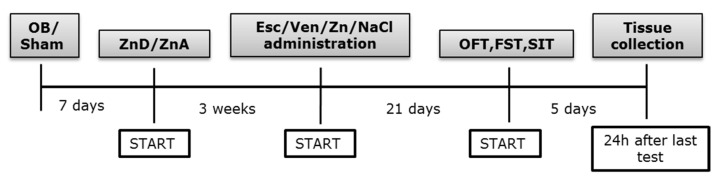
Experimental paradigm. Rats were subjected to bilateral olfactory bulbectomy, and sham-operated rats were similarly treated but with bulbs left intact. Rats were then fed a zinc adequate diet (ZnA, 50 mg Zn/kg) or a zinc-deficient diet (ZnD, 3 mg Zn/kg). At the beginning of the fourth-week of diet, animals were subjected to the treatment for 21 days and were further used for the behavioral tests or biochemical analysis. OB: olfactory bulbectomy model, Sham: operated rats with bulbs left intact, Esc: Escitalopram, Ven: Venlafaxine, OFT: open field test, FST: forced swim tests, SIT: sucrose intake test.

Twenty-four hours after the last dose of chronic drug injection (day 22), the open field test was carried out. After the test, animals were injected once again, and twenty-four hours later (day 23) the FST was carried out. After this test, animals were subjected to 2 days of a training session followed by the sucrose intake test on day 3 (the 26th day from treatment inception). During the training sessions, drugs were administered at intervals of twenty-four hours. After the sucrose intake test, animals were injected once more. Twenty-four hours later, tissue was collected (Scheme 1). Only rats with completely removed olfactory bulbs but without significant damage to the frontal cortex (as visually assessed post-sacrifice) and rats showing hyperactivity in the open field test were selected for further studies. We had few control groups, which was not much of an issue. Our interest was mainly the OB + ZnD rats. Thus, finally, the rats were divided into the following groups:

1. Sham + ZnA-rats were similarly treated as OB, but with bulbs left intact and fed a zinc adequate diet (ZnA) of 50 mg Zn/kg.
2. OB + ZnA-rats were subjected to bilateral olfactory bulbectomy and fed a zinc adequate diet (ZnA) of 50 mg Zn/kg.
3. Sham + ZnD-rats were similarly treated as OB, but with bulbs left intact and fed a zinc-deficient diet (ZnD) of 3 mg Zn/kg.
4. OB + ZnD-rats were subjected to bilateral olfactory bulbectomy and fed a zinc-deficient diet (ZnD) of 3 mg Zn/kg.

### 2.5. Open Field Test (OFT)

The open field test (OFT) [23] was done with an “open field” apparatus (an arena 90 cm in diameter, divided into 10 cm squares by faint yellow lines) and surrounded by a 75 cm high aluminum sheet. The OFT was carried out in a darkened room. A 60 W bulb 90 cm above the floor was used for illumination. During the test, animals were allowed to explore the open field for 3 min freely. Ambulation scores represent the number of sector lines crossed with all four paws.

### 2.6. Forced Swim Test (FST)

The forced swim test were carried out according to the method of Porsolt [24]. The rats were placed in glass cylinders (height 40 cm, diameter 20 cm) containing 15 cm of water, maintained at 25 °C. Two swim sessions were conducted: an initial 15 min pretest followed 24 h later by a 5 min test. Rats were removed from cylinders and returned to their home cages after both sessions. During the 5 min test, immobility time (in which rats were judged to be immobile when they remained floating passively in the water) was measured.

### 2.7. Sucrose Intake Test (SIT)

The sucrose intake test was performed as published by Doboszewska [25]. A training session preceded a test session. The training and the test sessions were conducted in-home cages equipped with two bottles; one bottle contained 1% sucrose solution and the other water. During the training session, the animals were trained to consume a 1% sucrose solution for 48 h. To prevent the possible effects of side preference, the positions of the bottles were switched every 12 h. Following the training session, sterile water was provided for 6 h and then food and water were withheld for the next 18 h. The test session was performed 24 h after the training session and lasted 1 h. The rats were given a choice between water and 1% sucrose solution. Sucrose intake was measured by weighing pre-weighed bottles at the end of the tests.

### 2.8. Tissue and Blood Collection

Animals were sacrificed by rapid decapitation 24 h after the last dose of drugs administration (Scheme 1). Brains were rapidly removed, with the PFC and Hp dissected on an ice-cold glass plate for biochemical analyses. Tissues were frozen in dry ice and stored at −80 °C until required. Whole brains were frozen on dry ice and stored at −80 °C until required to analyze intracellular and synaptic zinc levels. Blood was collected for zinc determination stored at −80 °C until analysis.

### 2.9. Analysis of Zinc

Samples were weighed, and then mineralized in a closed microwave system Magnum II (Ertec). Mineralization was carried out in three stages of 10 min each using Suprapur^®^ HNO_3_ (65%) and H_2_O_2_ (30%), at a power of 70% and 100%, respectively, maintaining the temperature of the device at 290 °C. After mineralization, the solutions were transferred to quartz evaporators and evaporated on a heating plate at 180 °C to remove excess reagents and water. The residue obtained after evaporation was quantitatively transferred to 10 mL volumetric flasks with four-times-distilled water. To analyze zinc, the standard solutions were used. Zinc analyze was made using the atomic absorption spectroscopy (AAS) method with flame atomization (AAS iCE3300 Thermo Scientific™ spectrophotometer, Waltham, MA, USA). The least squares calibration curve was determined for a concentration range of 0.1–1 μg/g. Zinc analysis was performed with the following parameters: wavelength 217.0 nm, width of slit 0.5 nm using acetylene as fuel. Each sample was analyzed in three independent replicates [26]

### 2.10. Zinpyr-1 Staining

Brain sections from Sprague Dawley rats (16 µm thickness) were prepared from fresh snap-frozen brains using a cryostat (Leica CM 1850, Leica Biosystems Nussloch GmbH, Nußloch, Germany) with the knife set at −22 °C. Three sections of the brain of the same animal were collected on one microscope slide. For staining, the slices were thawed for 20 min at RT, fixed with 4% paraformaldehyde solution and 4% sucrose in phosphate-buffered saline (PBS) at RT for 15 min, and rinsed with 0.01 M PBS solution. The sections were then incubated with a solution of a cell-membrane permeable Zn fluorescent probe, ZP-1 (Santa Cruz Biotechnology, Dallas, TX, USA, sc-213182) at a concentration of 5 μM for 1 h at RT. The sections were doubly stained with 4′,6-Diamidino-2-Phenylindole, Dihydrochloride (DAPI) (Sigma Aldrich, Saint Louis, MO, USA, D9542) at a concentration of 300 nM. Additional sections were treated with the membrane-permeable zinc chelator N,N,N’,N’-Tetrakis (2-pyridylmethyl) ethylenediamine (TPEN) (Santa Cruz Biotechnology, sc-200131), at a concentration of 10 μM, for 40 min, before staining with ZP-1 and DAPI. After staining, coverslips were mounted using VectaMount (Vector Labs, Newark, CA, USA) [27,28] Images were taken with a Nikon Eclipse E600 (Nikon, Tokyo, Japan) fluorescent microscope equipped with a camera (Leica Microsystems CMS, GmbH Germany, Wetzlar, Germany) connected to a computer running the Leica Application Suite (LAS) version 4.5 software. Low-magnification grayscale images were analyzed for the mean ZP-1 intensities using Image J ver. 1, (LOCI, University of Wisconsin-Madison, Madison, WI, USA).

### 2.11. Timm Staining

Brain sections (16 µm thickness) were prepared from fresh snap-frozen brains using a cryostat (Leica CM 1900) with the knife set at −22 °C. Three brain sections from the same animal were collected on a microscope slide. The sections were placed in a 4-L desiccator jar containing 100 mL 0.1% Na_2_S solution adjusted to pH 7.3 with 2N HCl, thus forming H_2_S gas. The jar was pumped into a vacuum for several minutes so that H_2_S gas from the solution could fill the air space. The sections were left under these conditions for 1–12 h, depending on the desired staining pattern. After sulfide treatment, the slices were washed in 0.1 M phosphate buffer (pH 7.4) three times, 3 min each at RT. The sections were then stained using a commercially available kit, FD Rapid TimmStain Kit (FD NeuroTechnologies, Inc, Columbia, MD, USA). The sections were placed in a fresh staining solution made by mixing Solutions A, B, C, and D and incubated for 50 min at 30 °C in the dark, slides were then rinsed in double distilled or MilliQ (Merck, Burlington, MA, USA) water for 3 min in the dark. Then, sections were dehydrated through a series of ethanol, cleared in xylene, and coverslipped mounted using VectaMount (Vector Labs, Newark, CA, USA) ([29,30]. Microphotographs were taken using a light microscope Nikon Eclipse E600 (Nikon, Tokyo, Japan), equipped with a black and white camera (Leica Microsystems CMS, GmbH Germany, Wetzlar, Germany) connected to a computer running the Leica Application Suite (LAS) version 4.5 software (GmbH Germany, Wetzlar, Germany).

### 2.12. Western Blot Analysis

Zinc transporter protein levels were assayed by Western blotting [30]. Hippocampal and prefrontal cortical tissue samples were sonicated in 2% sodium dodecyl sulfate solution (SDS) (10 mg/100 μL) and denatured at 95 °C for 10 min. Samples were centrifuged for 5 min at 10,000 rpm at 4 °C, and protein concentration in the supernatant was determined by the bicinchoninic acid assay method (Pierce). After denaturation, proteins were electrophoresed on a 10% SDS-polyacrylamide gel and transferred onto a nitrocellulose membrane (BioRad, Hercules, CA, USA). To prevent non-specific binding, membranes were processed in a 1% blocking solution (BM chemiluminescence Western Blotting Kit (Mouse/Rabbit) from Roche, Basel, Switzerland) followed by overnight incubation at 4 °C with antibodies of interest (rabbit polyclonal anti-ZnT1 antibody (1:1000, Synaptic System); mouse monoclonal anti-ZnT3 (1:1000, Synaptic System, Göttingen, Germany). Antibodies were diluted in a 0.5% blocking reagent (Roche). Membranes were then washed three times (10 min) in Tris-buffered saline containing Tween (TBS-T), followed by incubation in secondary anti-mouse/rabbit-IgG-peroxidase conjugated (1:7000, Roche) for 60 min. After that, membranes were washed three times (10 min) with TBS-T and incubated with a detection reagent (Roche). Proteins on the membranes were visualized and quantified using the Fuji-Las 1000 system and Fuji Image Gauge v.4.0 software (Fuji, Tokyo, Japan). GAPDH (mouse monoclonal anti-GAPDH antibody; diluted 1:500; Millipore, Merck, Burlington, MA, USA) served as transfer and loading control. Results are expressed as the ratio of the optical density of a particular protein to the optical density of GAPDH on the same blot.

### 2.13. Statistical Analysis

Data were analyzed using the GraphPad Prism software (ver. 4.0, San Diego, CA, USA). The results are expressed as group means ± SEM. Behavioral data were analyzed using a two-way ANOVA and the Newman–Keuls multiple comparisons tests or one-way ANOVA and the Newman–Keuls multiple comparisons tests). Biochemical data were analyzed using a two-way ANOVA and the Newman–Keuls multiple comparisons test or one-way ANOVA and the Newman–Keuls multiple comparisons test. Significance was assumed at *p* < 0.05.

## 3. Results

### 3.1. Behavioral Studies

#### 3.1.1. The Effect of OB, ZnD, and OB + ZnD Model on the Behavior of Rats

**Figure 1:**
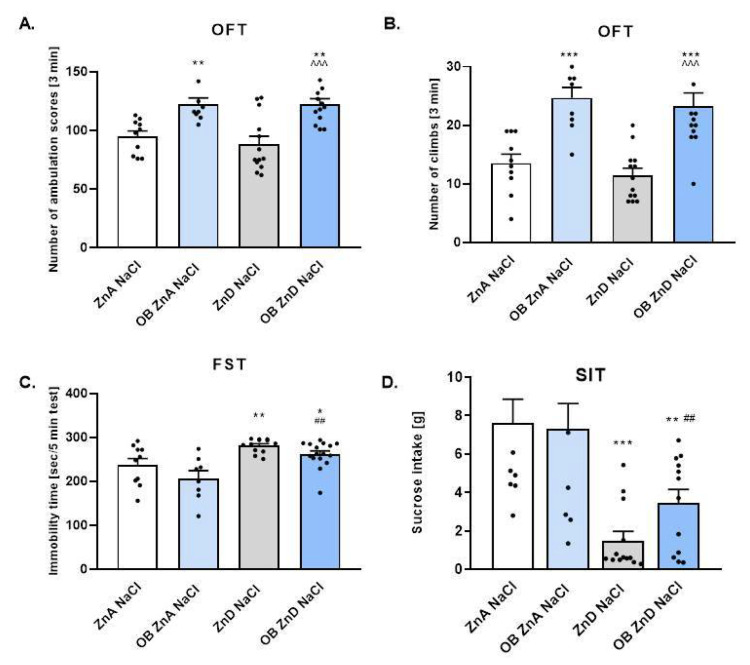
The effect of OB, ZnD, and OB + ZnD models on the behavior of rats in the open field test (OFT, (**A**,**B**)), forced swim test (FST, (**C**)), and sucrose intake test (SIT, (**D**)). Data were analyzed using a two-way ANOVA and the Newman–Keuls multiple comparisons test, * *p* < 0.05, ** *p* < 0.01, *** *p* < 0.001 vs. ZnA NaCl; ^^^ *p* < 0.001 vs. ZnD NaCl; ## *p* < 0.01 vs. OB ZnA NaCl. Values are expressed as mean ± SEM (*n* = 8–15). OB: olfactory bulbectomy model, ZnD: zinc-deficient diet, and OB + ZnD: olfactory bulbectomy model and zinc-deficient diet.

All rats used in the present studies were subjected to surgery. The control group described on the graph as ZnA + NaCl was also subjected to surgery; however, their olfactory bulbs were left intact. The ZnD + NaCl rats were similarly treated. Rats from the OB and OB + ZnD groups had their olfactory bulbs removed. This paradigm was used throughout the whole study. The OFT was carried out to determine the effects of OB, ZnD, and OB + ZnD procedures on rat behavior. As shown in Figure 1A, OB induced hyperactivity in rats (*p* < 0.01); ZnD alone did not affect rat activity (*p* > 0.05). However, rats subjected to OB and ZnD simultaneously exhibited increased activity compared to the control group (*p* < 0.01) and ZnD group (*p* < 0.001). The other parameter measured in the OFT was the number of climbs. As shown in Figure 1B rats, subjected to the OB procedure and OB + ZnD exhibited an increased number of climbs (*p* < 0.001). ZnD alone did not influence the number of climbs (*p* < 0.05). The OFT is a commonly used test for measuring hyperactivity induced by the OB model, which is considered a model of agitated depression. The other tests used to evaluate depressive like-behavior are the FST and SIT, which measure anhedonia. In our studies, ZnD and OB + ZnD induced statistically significant changes in the immobility time of rats in the FST (*p* < 0.01 and *p* < 0.05, respectively) compared to the control group (Figure 1C). On the other hand, we found that ZnD and OB + ZnD procedures induced anhedonia in rats (Figure 1D), observed as a decrease in sucrose intake (*p* < 0.001 and *p* < 0.01, respectively). Two-way ANOVA analysis: OFT: [1A: no significant interaction (F(1, 41) = 0.3120, *p* = 0.5795; no effect of ZnD F(1, 41) = 0.3468, *p* = 0.5592, significant effect of OB F(1, 41) = 29.72, *p* < 0.0001; 1B: no significant interaction F(1, 41) = 0.03591, *p* = 0.8506; no effect of ZnD F(1, 41) = 0.8977, *p* = 0.3490; significant effect of OB F(1, 41) = 41.19, *p* < 0.0001]; FST [1C: no significant interaction F(1, 41) = 0.0042 *p* = 0.9484; significant effect of ZnD F(1, 41) = 26.48, *p* < 0.0001; no effect of OB F(1, 41) = 2.848, *p* = 0.0991]; SIT: [1D: no significant interaction F(1, 41) = 1.423, *p* = 0.2398; significant effect of ZnD F(1, 41) = 28.07, *p* < 0.0001; no effect of OB F(1, 41) = 0.7801, *p* = 0.3822].

#### 3.1.2. The Effect of Antidepressants and Zn Supplementation on Rats Subjected to the OB + ZnD Model

**Figure 2:**
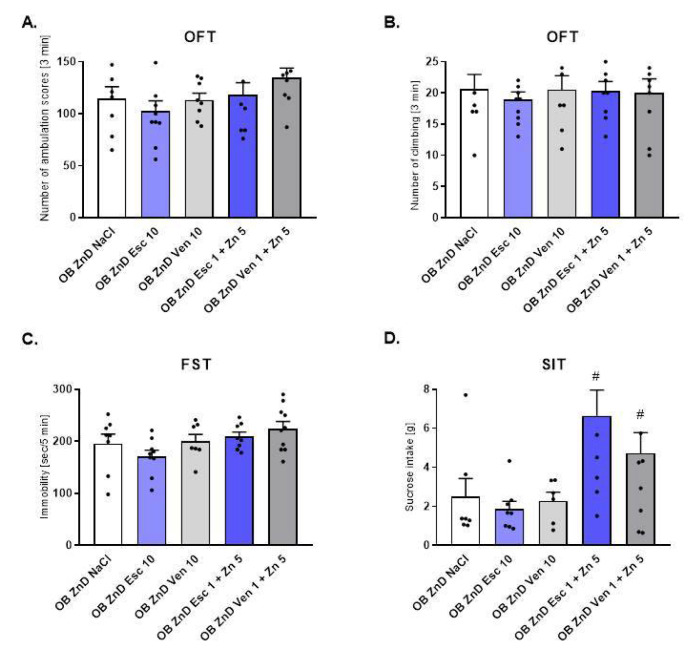
The effects of antidepressants (Esc (Escitalopram), Ven (Venlafaxine)) and Zn supplementation on rats subjected to the OB + ZnD model in the OFT (**A**,**B**), FST (**C**), and SIT (**D**). Data were analyzed using a one-way ANOVA and the Newman–Keuls multiple comparisons test. # *p* < 0.05 vs. OB ZnD NaCl. Values are expressed as mean ± SEM (*n* = 7–10).

To ascertain whether ZnD induces resistance to antidepressants we evaluated the effect of two different antidepressant drugs: escitalopram (Esc) and venlafaxine (Ven). As shown in Figure 2A,B, neither chronic treatment with 10 mg/kg of Esc nor 10 mg/kg of Ven influenced rat behavior in the OFT [2A: F(4, 39) = 1.465, *p* = 0.2313; 2B: F(4, 39) = 0.1414, *p* = 0.9657] and FST (*p* > 0.05) [2C: F(4, 37) = 2.272, *p* = 0.0799] compared to OB + ZnD rats. Similarly, chronic treatment with lower doses of Esc and Ven (1 mg/kg) and Zn (5 mg/kg) did not alter rat activity in the OFT (2A, B) and FST (2C) (*p* > 0.05). In the SIT (2D) chronic treatment with Esc and Ven (1 mg/kg) plus Zn (5 mg/kg) increased sucrose intake (*p* < 0.05) compared to NaCl treated OB + ZnD rats [2D: F(4, 36) = 3.274, *p* = 0.0218].

#### 3.1.3. The Effects of OB, ZnD, and OB + ZnD Model on Serum Zinc Levels

To assay for the effects of the OB, ZnD, and OB + ZnD procedures on serum zinc levels, the AAS was performed. As shown in Table 1, OB alone did not alter serum zinc levels when compared to the ZnA NaCl group. On the other hand, the ZnD and OB + ZnD models significantly decreased serum zinc levels (*p* < 0.001) compared to the ZnA NaCl and ZnA OB NaCl groups respectively. A two-way ANOVA showed significant effects of ZnD [F(1, 17) = 19.25; *p* = 0.0004]; non-significant effects of OB NaCl [F(1, 17) = 0.2234; *p* = 0,6425 and non-significant interaction [F(1, 17) = 2.108; *p* = 0.1648].

#### 3.1.4. The Effects of Antidepressants and Zn Supplementation on Serum Zinc Levels in Rats Subjected to the OB + ZnD Model

To describe the effects of antidepressants (Esc, Ven) and Zn supplementation procedures on serum zinc levels, the AAS was performed. As shown in Table 2, chronic treatment with lower doses of Esc and Ven (1 mg/kg) and Zn (5 mg/kg) increased serum zinc levels [F(4, 15) = 18.45, *p* < 0.0001] compared to the OB + ZnD NaCl group. Chronic treatment with 10 mg/kg of Esc or Ven did not significantly alter the earlier increase in serum zinc levels (*p* > 0.05).

#### 3.1.5. The Effects of Antidepressants and Zn Supplementation on Intracellular Zinc Concentration in Rats Subjected to the OB + ZnD Model

**Figure 3:**
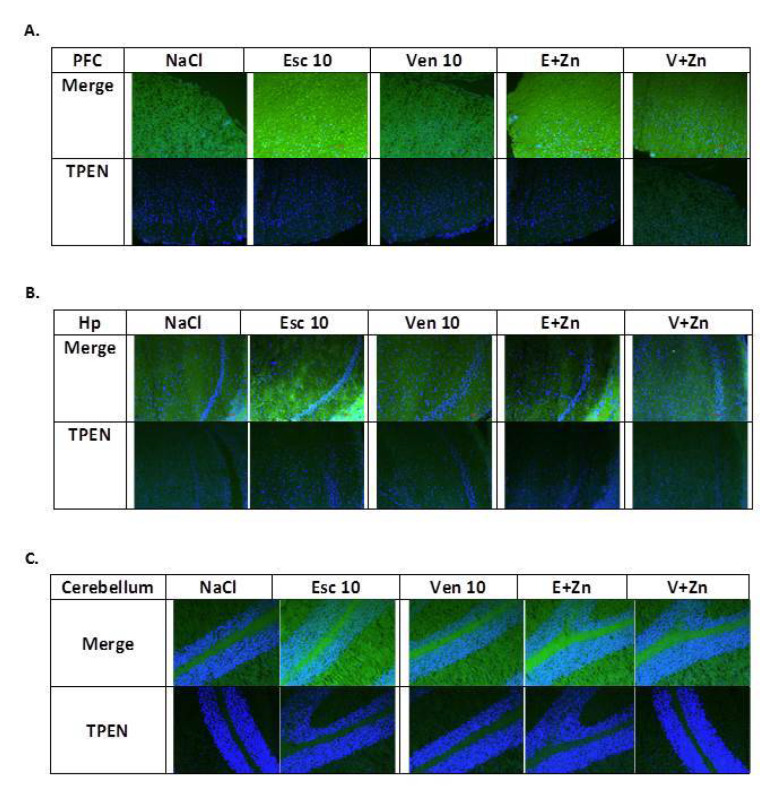
The representative images of the effects of antidepressants (Esc, Ven) and Zn supplementation on intracellular zinc concentration in the prefrontal cortex (PFC, (**A**)) and hippocampus (Hp (**B**)), and cerebellum (**C**) of rats subjected to the OB + ZnD model using fluorescent Zinpyr-1 (ZP-1) staining. Sections were double-stained with DAPI (blue) to reveal cell nuclei and ZP-1 (green) to detect fluorescent intracellular Zn^2+^. Merged images include ZP-1 and DAPI (upper panel) or TPEN/ZP-1 and DAPI (lower panel) in the PFC (**A**), Hp (**B**), and cerebellum (**C**). ZP-1 fluorescence is mostly absent in TPEN-treated sections, indicating high specificity of ZP-1 for Zn^2+^. Scale bar = 50 μM.

To assess whether antidepressant treatment and Zn supplementation in OB + ZnD rats leads to alterations in Zn^2+^ levels in selected brain structures, we visualized Zn^2+^ using Zinpyr-1 in rat brain sections (Figure 3A–C). The specificity of the signal was achieved by the application of the Zn^2+^ chelator, TPEN (Figure 3A–C lower panel).

As shown in Table 3, chronic treatment with 10 mg/kg of Esc and Esc (1 mg/kg) and Zn (5 mg/kg), but not with 10 mg/kg of Ven or Ven (1 mg/kg) and Zn (5 mg/kg) increased intracellular zinc levels in the PFC [F(4, 13) = 3.591, *p* = 0.0350] compared to the OB ZnD NaCl group. In cerebellum chronic treatment with 10 mg/kg of Esc, Ven (1 mg/kg), and Zn (5 mg/kg) and Esc (1 mg/kg) and Zn (5 mg/kg), but not with 10 mg/kg Ven increased intracellular zinc levels [F(4, 14) = 6.193, *p* = 0.0044] compared to the OB ZnD NaCl group. In Hp, we did not observe any alterations after chronic treatment with antidepressants and Zn supplementation [F(4, 18) = 1.355, *p* > 0.05].

#### 3.1.6. The Effects of Antidepressants and Zn Supplementation on Brain Synaptic Zinc Levels in Rats Subjected to the OB + ZnD Model

**Figure 4:**
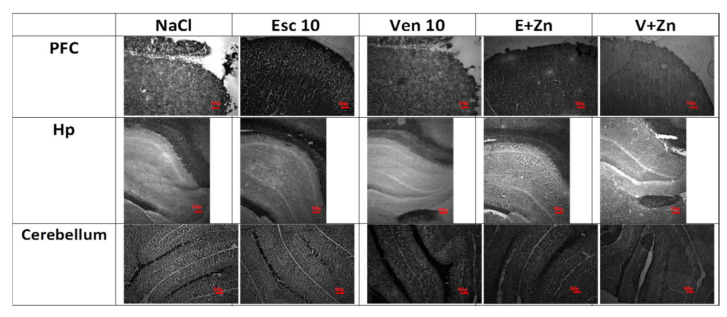
The representative images of the effects of antidepressants (Esc, Ven) and Zn supplementation on synaptic zinc levels in the prefrontal cortex (PFC), hippocampus (Hp), and cerebellum of rats subjected to the OB + ZnD model (Timm staining). Scale bar = 50 μM.

To assess whether antidepressant treatment and Zn supplementation in OB + ZnD rats leads to alterations in brain synaptic zinc levels in selected brain structures, we visualized Zn^2+^ using Timm staining in rat brain sections (Figure 4).

As shown in Table 4, chronic treatment with 10 mg/kg of Esc and Esc (1 mg/kg), Zn (5 mg/kg), and 10 mg/kg Ven or Ven (1 mg/kg) and Zn (5 mg/kg) increased synaptic zinc levels in the PFC [F(4, 14) = 5.616; *p* = 0.124] compared to the OB ZnD NaCl group. In the cerebellum, chronic treatment with 10 mg/kg of Ven; Esc (1 mg/kg) and Zn (5 mg/kg) and Ven (1 mg/kg) and Zn (5 mg/kg), but not Esc increased synaptic zinc levels [F(4, 19) = 9.697; *p*= 0.0004] compared to the OB ZnD NaCl group. In the Hp we did not observe any effects after chronic treatment with antidepressants and Zn supplementation [F(4, 11) = 0.1760; *p* > 0.05].

#### 3.1.7. The Effects of OB, ZnD, and OB + ZnD Models on the Levels of ZnT1 and ZnT3 in the PFC and Hp

**Figure 5:**
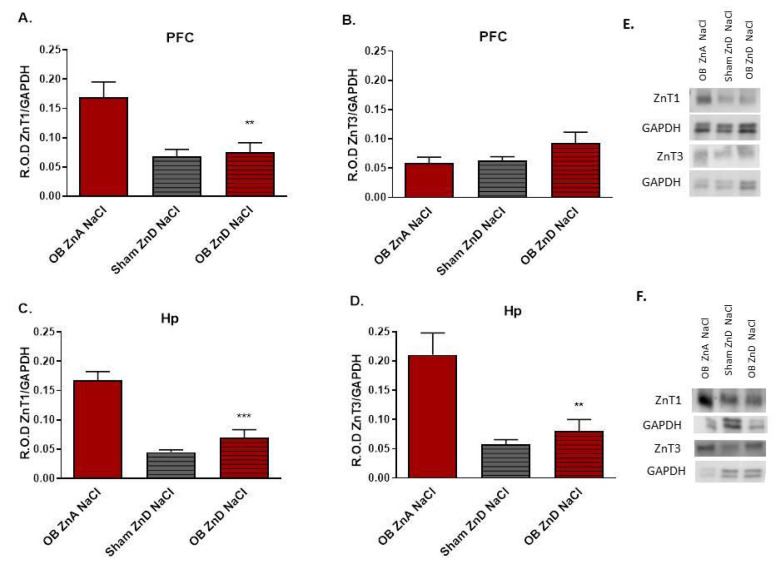
The effects of OB, ZnD, and OB + ZnD models on the zinc transporter protein levels in the prefrontal cortex (PFC, (**A**,**B**)) and hippocampus (Hp, (**C**,**D**)). Representative blots for PFC (**E**); representative blots for Hp (**F**). Data were analyzed using a one-way ANOVA and the Newman–Keuls multiple comparisons test. Values are expressed as mean ± SEM (*n* = 6–7). OB: olfactory bulbectomy model, ZnD: zinc-deficient diet, OB + ZnD: olfactory bulbectomy model and zinc-deficient diet GAPDH: glyceraldehyde-3-phosphate dehydrogenase, R.O.D ZnT1: relative optical density of zinc transporter 1, R.O.D ZnT3: relative optical density of zinc transporter 3. ** *p* < 0.01; *** *p* < 0.0001.

Western blot analysis showed decreased levels of ZnT1 protein in the PFC (F(2, 15) = 9.537; *p* < 0.01) and Hp (F(2, 13) = 27.20; *p* < 0.0001) (Figure 5A,C) of rats subjected to the OB + ZnD model vs. rats subjected only to the OB procedure. However, there were no differences in ZnT1 protein levels between OB + ZnD and ZnD treated rats (Figure 5A,C). The OB + ZnD procedure also decreased levels of the ZnT3 protein in the Hp (F(2, 13) = 12.98; *p* < 0.001) vs. OB ZnA (Figure 5D) with no effect in the PFC (F(2, 14) = 2.210 (Figure 5B).

#### 3.1.8. The Effect of Antidepressants and Zn Supplementation on the ZnT1 and ZnT3 Protein Levels in Rats Subjected to the OB + ZnD Model

**Figure 6:**
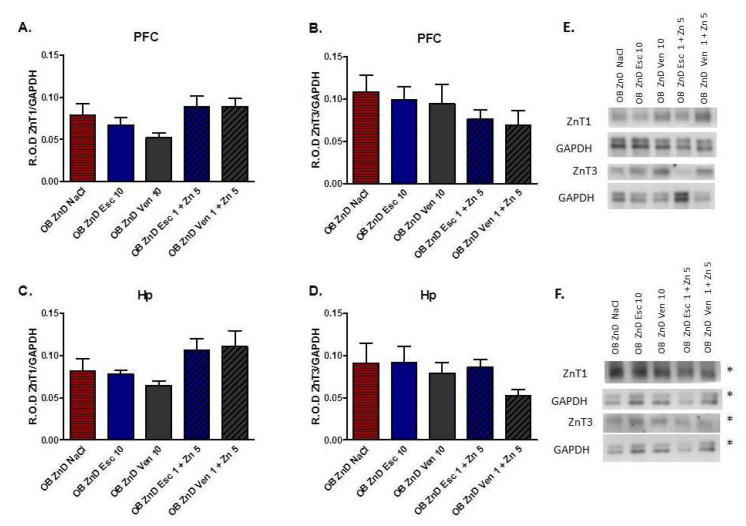
The effects of antidepressants (Esc, Ven) and Zn supplementation on zinc transporter protein levels in the prefrontal cortex (PFC, (**A**,**B**)) and hippocampus (Hp, (**C**,**D**)) of rats subjected to the OB + ZnD model. Representative blots for PFC (**E**); representative blots for Hp [(**F**), * the images are from the same membrane]. Data were analyzed using a one-way ANOVA and the Newman–Keuls multiple comparisons test. Values are expressed as mean ± SEM (*n* = 6–7).

As shown in Figure 6A–D, neither chronic treatment with 10 mg/kg of Esc or Ven nor lower doses of Esc and Ven (1 mg/kg) and Zn (5 mg/kg) induced significant effects on protein levels (*p* > 0.05) of ZnT1 [F(4, 28) = 2.259; *p* = 0.3615] and ZnT3 [F(4, 25) = 0.6884; *p* = 0.6068] in the PFC of OB + ZnD rats (Figure 6A,B). Similarly, in the Hp, chronic treatment with 10 mg/kg of Esc or Ven nor with lower dose of Esc and Ven (1 mg/kg) and Zn (5 mg/kg) did not alter protein levels (*p* > 0.05) of ZnT1 [F(4, 27) = 2.694; *p* = 0.0521] and ZnT3 [F(4, 30) = 0.9430; *p* = 0.4527] compared to the OB + ZnD NaCl group in Hp (Figure 6C,D).

## 4. Discussion

The present research aimed to ascertain the possibility of modeling drug-resistant depression in animals and whether zinc deficiency could trigger drug resistance. To induce depressive-like behavior, rats were subjected to the well-known OB model of depression and 3 weeks of zinc restriction [16,17,30,31]. The OB model is a model of agitated depression [21], and the primary behavioral change induced by OB is increased activity [9,10,30]. We reproduced hyperactivity in OB rats following the increased number of lines and climbs observed in the OFT. On the other hand, ZnD did not cause any significant changes in the behavior of rats in the OFT. However, rats subjected to the OB + ZnD model displayed hyperactivity in the OFT, indicating that the elevated activity observed in rats subjected to OB + ZnD is related to the OB effect and not ZnD [9,10,30]. The FST is commonly used as a rodent screening test for evaluating the antidepressant-like activity of compounds; recently, this test has also been used to measure depressive-like effects in rodent models of depression. In our studies, OB did not alter rat behavior in the FST. In the FST, depressive-like behavior is observed as increased immobility time. Thus, compounds and procedures that influence the locomotor activity of rodents may give false positive or negative effects. This might apply to rats subjected to the OB procedure, which induces hyperactivity in animals [9,10]. On the other hand, we observed increased immobility time in the ZnD rats compared to ZnA control rats and a similar effect with the OB + ZnD rats. This indicates that the depressive-like effects induced by OB + ZnD in the FST are related to the ZnD effect, not OB. To further confirm this, the SIT was performed. Rats subjected to ZnD exhibited anhedonia (measured as a decrease in sucrose intake). On the other hand, there were no changes in sucrose intake with OB rats. Several studies in rodents indicate that ZnD induces depressive-like symptoms, including anhedonia [15,16,17,32,33,34]. Our results thus confirm that anhedonia observed in the OB + ZnD model is attributable to the effect of ZnD, not OB.

In the next step, we examined the effects of chronic treatment with selective serotonin reuptake inhibitor (SSRI) escitalopram (Esc), and serotonin–norepinephrine reuptake inhibitor (SNRI) venlafaxine (VEN), or a combined antidepressant treatment (Esc/Ven inactive dose) and Zn on OB + ZnD induced alterations. Our results showed that depression-like behavior caused by the OB + ZnD model is sensitive to chronic (3-weeks) treatment with Esc in the OFT but not FST or SIT. In randomized and controlled studies involving patients with moderate or severe depression, Esc (10, 20 mg/kg) was more effective than placebo and comparable or more effective than citalopram in ameliorating depressive symptoms [35,36]. In animals, chronic Esc administration reversed OB-induced behavior in the OFT, social interaction, and hypermotionality tests in rats [37]. Another study showed that chronic administration of Esc normalized OB-induced hyperactivity in rats, and this effect lasted up to 10 weeks after treatment cessation and in a dose-dependent manner [38]. In our studies chronic treatment of Esc (10 mg/kg) also reversed the OB induced hyperactivity in OFT in rats (Figure S1). As we mentioned above, hyperactivity observed in the OB + ZnD model was attributable to the OB effect; therefore, the positive action of Esc in this model is justified.

Esc has not been previously tested in a model of ZnD in rats. Notwithstanding, Doboszewska et al. (2016) reported that chronic (2 weeks) treatment with fluoxetine (selective serotonin reuptake inhibitor (SSRI)) reduces immobility time in the FST after a 4-week ZnD diet. On the contrary, Tassabehji et al. (2008) reported that chronic (3 weeks) treatment with fluoxetine did not reduce immobility time in the FST in ZnD rats. However, Tassabehji et al. (2008) did not observe depression-like behavior (increased immobility time) in ZnD rats compared to control, possibly because of the shorter duration of the diet in the study [33]. As we indicated above, the depressive effect in the OB + ZnD rats observed in the FST is the effect of ZnD. The absence of an Esc effect in this test may indicate that ZnD may be responsible for inducing drug resistance.

Venlafaxine (Ven) is another drug with exhibited potential in depression, especially treatment-resistant depression. Preclinical studies indicate Ven is effective in animal models of depression. Chronic administration of Ven reversed depressive-like behavior caused by OB in mice in the TST [39]. Ven also normalized changes after OB in rats in the OFT [40]. Our data also showed positive effects of Ven (10 mg/kg/21 days) in the OFT in OB rats (Figure S1). However, Ven did not reverse the OB + ZnD-induced hyperactivity in rats, indicating that our model might be resistant to Ven treatment. So far, Ven has not been studied in ZnD.

Interestingly, depressive-like symptoms in the SIT were reversed by chronic Esc/Ven (at low dose) and Zn, but not Esc or Ven at a higher dose (10 mg/kg), while both Esc and Ven given chronically (10 mg/kg/21 days) increased sucrose intake in OB rats (Figure S2). Anhedonia, developed mainly due to ZnD in our model, is easily reversed when antidepressants are co-administered with Zn. Therefore, it makes sense to augment antidepressant therapy with zinc in cases where depression is accompanied by zinc deficiency.

Overall, clinical studies have indicated that zinc supplementation could be an adjunct to increasing the effectiveness of tricyclic antidepressants and selective serotonin reuptake inhibitors but not selective noradrenaline reuptake inhibitors [41,42]. The beneficial effect of zinc monotherapy in relieving depressive symptoms was reported by Solati et al. (2015), indicating that zinc may play an important role in treating depression. Zinc induced an antidepressant-like effect (reduction in immobility time) in the FST and tail suspension test (TST) [43,44,45]. Zinc was also active in different models of depression, such as OB [45], chronic mild stress (CMS) model of depression [46], and chronic unpredictable stress (CUS) [47].

Zinc Concentration in Serum/Brain

Several studies have shown that human depression is accompanied by decreased serum Zn levels. Reduced serum zinc concentration has been found in the patients suffering from major depressive disorder, recurrent major depressive disorders, and depressed patients with end-stage renal disease undergoing hemodialysis [13,48]. Moreover, patients suffering from treatment-resistant depression exhibited much lower serum zinc concentrations than their non-treatment-resistant depressed counterparts [14].

Reduced serum zinc levels are also typical in rats subjected to chronic ZnD. After 6 weeks of a zinc-deficient diet (ZnD, 3 mg Zn/kg), significant reductions in serum zinc levels were observed in rats [17]. Similarly, our present studies showed decreased serum zinc levels in rats subjected to 6 weeks of ZnD. Interestingly, the same effect was observed in rats subjected to the OB + ZnD model, albeit no effect was seen in OB by itself. No changes in serum zinc levels were present in the chronic mild stress (CMS) model [49]. Based on the above, decreased serum zinc level seems to be a biomarker of human depression and our studies indicate that the OB + ZnD model exhibits similarities to human depression concerning zinc.

Clinical studies showed that decreased serum zinc levels in depression were normalized after chronic imipramine treatment with or without zinc supplementation [14]. However, treatment with trazodone alone or combined with fluoxetine and pindolol did not induce significant changes in zinc levels. Of note, treatment-resistant depression represented a substantial percentage of the studies demonstrating a lack of normalization of serum zinc concentration. Preclinical studies showed that chronic treatment with citalopram and fluoxetine, but not imipramine or ECS, increased serum zinc levels in rats [50]. Furthermore, antidepressant or Zn treatment normalized stress-induced or zinc deficiency-induced reduction in serum zinc in rodents [17,51,52]. In the present studies, decreased serum zinc levels in the OB + ZnD group were reversed by a combined treatment of low dose Esc and Ven supplemented with zinc.

Recent studies showed that the administration of Zn + IMI normalizes CRS-induced changes not only in serum but also in the brain (Hp) Zn levels [53]. Chronic administration of hyperforin increased zinc concentration in the Hp of mice. Chronic ECS treatment greatly enhanced zinc levels in the Hp and slightly in the cortex and cerebellum. In contrast, chronic treatment with citalopram or imipramine slightly increased zinc levels only in the Hp [50]. These data suggest a dynamic redistribution of Zn ions after antidepressant treatment. These studies indicated changes in total zinc levels in the different brain structures.

However, the mechanisms of such an effect are still unknown. We have previously shown that IMI enhances Zn transfer through the intestinal tract and influences its redistribution between the blood and brain [54]. However, it is unclear which brain Zn pool (total, intracellular, or synaptic) might be affected the most.

Only one study so far showed that chronic treatment with citalopram (20 mg/kg) but not imipramine (20 mg/kg) increased the pool of presynaptic zinc in the rat prefrontal cortex [55]. However, we cannot exclude that antidepressants might also influence other Zn pools. To explain this hypothesis, we measured the intracellular and synaptic Zn levels in several brain structures in rats subjected to the OB + ZnD model and Esc or Ven treatment. Our unpublished data showed a reduction in intracellular and synaptic zinc levels in the PFC and cerebellum of rats subjected to ZnD and OB + ZnD procedures. We did not observe any changes in Zn pools in the Hp (data not shown). Esc at a higher dose alone and in combination with a low dose of Zn increased the intracellular and synaptic Zn concentrations in the PFC. On the other hand, Ven at a higher dose and in combination with a low dose of Zn increased only synaptic Zn in the PFC.

Interestingly, our studies indicated perturbed intracellular and synaptic Zn levels after antidepressant treatment in the cerebellum of rats subjected to OB + ZnD model. We selected the cerebellum for our research for two reasons. First, it is one of the few brain structures with a high zinc concentration, and second, cerebellar disorders can be the basis of depressive disorders. Several clinical studies indicate that autism, schizophrenia, depression, bipolar disorder or anxiety disorders, and PTSD may be caused by cerebellar disorders [56]. Initial studies carried out by Soares and Mann (1997) led to conclusions about the cerebellar basis of depression due to its reduced total volume in patients with unipolar depression. One of the diseases first associated with depressive disorders was the degenerative disease of the cerebellum [57].

Depression is also often accompanied by psychomotor symptoms associated with disturbances in gait, posture, and motor coordination characteristic of cerebellar ataxia [58]. The association of ataxia with impaired serotonin secretion through selective serotonin reuptake inhibitors (SSRIs) led to conclusions about the disappearance of depressive symptoms in patients with cerebellar ataxia [59], further linking the cerebellum to depression.

Because ZnT-3 is expressed in the cerebellar cortex, there is a likelihood that zinc is involved in cerebellar synaptic transmission. On the other hand, zinc deficiency could decrease GABA concentrations in the cerebellum. Consequently, zinc deficiency may lead to cerebellar deficits since GABA is the primary neurotransmitter in the cerebellar Purkinje cells [60]. Additionally, the mouse cerebellum expresses high levels of ZnT-1, 3, 4, and 6 through the Bergman glial cells in the Purkinje cell layer [61], suggesting these cells may play an essential role in cerebellar zinc homeostasis.

No changes were observed in the Hp, suggesting that the Hp is more resistant to Zn dyshomeostasis than the PFC. The hippocampus, especially the CA3 field and the dentate gyrus, contains large amounts of zinc [62,63], which can serve as “reserve pools” when zinc is deficient. Therefore, intracellular and synaptic Zn may be resistant to manipulations like OB + ZnD or antidepressant and/or Zn supplementation. A 4-week ZnD diet resulted in a reduction in the extracellular Zn^2+^ pool in the Hp [64]. We can assume from our studies that a compensatory mechanism in the Hp keeps zinc levels constant in this structure, with the PFC Zn pool more vulnerable to insults.

Changes in extracellular and intracellular Zn concentrations contribute to Zn signaling, and consequently, to physiological and pathological processes [62]. Zinc homeostasis is controlled by different proteins involved in the uptake, excretion, and intracellular storage/trafficking of Zn. Thus, we examined the expression of proteins involved in maintaining zinc homeostasis in the brain in the next step. For our studies, we chose highly expressed ZnTs in the brain for which changes in expression and distribution during pathophysiological processes have been demonstrated [65,66,67,68]. Whitfield et al. (2015) showed a significant reduction in ZnT3 protein levels in the PFC of depressed patients with dementia. Our recent post-mortem studies showed an increase in ZnT1, ZnT4, and ZnT5 in the PFC of MDD subjects relative to controls, while ZnT3 protein level was decreased in MDD [69]. Preclinical studies in OB rats showed increases in ZnT1 protein levels in the PFC and Hp, decreased ZnT3 in the PFC, and no changes in ZnT4, ZnT5, and ZnT6 in the PFC and Hp. Chronic amitriptyline treatment did not influence ZnT protein levels in sham and OB rats [30].

Our present studies showed a significant decrease in ZnT1 protein levels in the PFC and Hp of OB + ZnD rats compared with animals that received a zinc-adequate diet and were subjected to OB. Similarly, we observed dysregulation of ZnT3 in the Hp with no changes in the PFC. Chronic administration of Esc/Ven or Esc/Ven (inactive dose) and Zn had no effects on ZnT levels in the PFC or Hp.

## 5. Conclusions

The behavioral part of our research indicates that the ZnD does not produce additive effects, in terms of the level of depressive symptoms, in OB rats. However, the results of OFT, FST, and SIT show that the antidepressant effect of the drugs used (Esc, Ven) is blunted under conditions of zinc deficiency. The behavioral changes observed in SIT were reversed when Esc and Ven were co-administered with zinc. Behavioral changes induced by Esc/Ven +Zn correlated with increased serum zinc levels and in part with increased levels of intracellular and synaptic zinc in selected brain structures of OB + ZnD rats. These results provide additional confirmation that zinc deficiency may be a variable determining the occurrence of drug-resistant depression.

## Figures and Tables

**Table 1:** The effects of OB, ZnD, and OB + ZnD models on serum zinc levels measured by flame atomic absorption spectrometry.

Treatment	Mean ± SEM
ZnA NaCl	0.3580 ± 0.082
ZnA OB NaCl	0.4945 ± 0.130
ZnD NaCl	0.1497 ± 0.027 *
ZnD OB NaCl	0.0803 ± 0.014 ^##^

Data were analyzed using a two-way ANOVA followed by the Newman–Keuls multiple comparisons tests; * *p* < 0.05 vs. ZnA, ^##^ *p* < 0.001 vs. ZnA + OB. (*n* = 3–7). ZnA: zinc adequate diet, ZnD: zinc deficient diet, OB: olfactory bulbectomy model

**Table 2:** The effects of antidepressants (Esc, Ven) and Zn supplementation on serum zinc levels in rats subjected to the OB + ZnD model.

Treatment	Mean ± SEM
OB ZnD NaCl	0.336 ± 0.064
OB ZnD Esc 10	0.348 ± 0.077
OB ZnD Ven 10	0.265 ± 0.126
OB ZnD Esc 1 + Zn 5	0.578 ± 0.099 ***
OB ZnD Ven 1 + Zn 5	0.517 ± 0.084 **

Data were analyzed using a one-way ANOVA and the Newman–Keuls multiple comparisons test; ** *p* < 0.01; *** *p* < 0.0001. Values are expressed as mean ± SEM (*n* = 4).

**Table 3:** The effects of antidepressants and Zn supplementation on intracellular zinc concentration in rats subjected to the OB + ZnD model using fluorescent Zinpyr-1 staining.

Brain Structure	Treatment	Mean ± SEM
	OB ZnD NaCl	54.056 ± 8.514
	OB ZnD Esc 10	111.57 ± 9.431 *
PFC	OB ZnD Ven 10	85.41 ± 3.895
	OB ZnD Esc 1 + Zn 5	110.87 ± 8.837 *
	OB ZnD Ven 1 + Zn 5	81.18 ± 6.010
	OB ZnD NaCl	107.2 ± 11.234
	OB ZnD Esc 10	98.12 ± 8.271
Hp	OB ZnD Ven 10	88.66 ± 8.505
	OB ZnD Esc 1 + Zn 5	87.38 ± 8.023
	OB ZnD Ven 1 + Zn 5	90.04 ± 9.042
	OB ZnD NaCl	59.58 ± 5.401
	OB ZnD Esc 10	119.66 ± 8.068 **
Cerebellum	OB ZnD Ven 10	114.0 ± 8.521 *
	OB ZnD Esc 1 + Zn 5	119.75 ± 10.276 *
	OB ZnD Ven 1 + Zn 5	79.16 ± 10.510

Data were analyzed using a one-way ANOVA and the Newman–Keuls multiple comparisons test; * *p* < 0.05; ** *p* < 0.001 (*n* = 3–5).

**Table 4:** The effects of antidepressants and Zn supplementation on synaptic zinc level in rats subjected to the OB + ZnD model using Timm staining.

Brain Structure	Treatment	Mean ± SEM
	OB ZnD NaCl	155.66 ± 7.48
	OB ZnD Esc 10	201.50 ± 5.05 **
PFC	OB ZnD Ven 10	197.44 ± 8.27 *
	OB ZnD Esc 1 + Zn 5	195.25 ± 7.68 **
	OB ZnD Ven 1 + Zn 5	183.66 ± 5.30 *
	OB ZnD NaCl	157.70 ± 13.11
	OB ZnD Esc 10	161.66 ± 13.19
Hp	OB ZnD Ven 10	153.25 ± 11.08
	OB ZnD Esc 1 + Zn 5	174.03 ± 13.92
	OB ZnD Ven 1 + Zn 5	150.27 ± 13.32
	OB ZnD NaCl	155.0 ± 9.78
	OB ZnD Esc 10	177.83 ± 6.83
Cerebellum	OB ZnD Ven 10	192.80 ± 6.32 **
	OB ZnD Esc 1 + Zn 5	204.22 ± 2.61 **
	OB ZnD Ven 1 + Zn 5	215.58 ± 8.76 ***

Data were analyzed using a one-way ANOVA and the Newman–Keuls multiple comparisons test; * *p* < 0.05; ** *p* < 0.01, *** *p* < 0.001.

## Data Availability

Data that support the findings of this study are available from the corresponding author upon reasonable request.

## Supplementary Materials

The following are available online at https://www.mdpi.com/article/10.3390/nu14132746/s1, Figure S1: The effect of antidepressants in rats subjected to the OB model on the behaviour in OFT, Figure S2: The effect of antidepressants in rats subjected to the OB model on the behaviour in SIT.

## References

[1] Busfield J. (2012). Challenging claims that mental illness has been increasing and mental well-being declining. Soc. Sci. Med..

[2] Hidaka B.H. (2012). Depression as a disease of modernity: Explanations for increasing prevalence. J. Affect. Disord..

[3] Souery D., Oswald P., Massat I., Bailer U., Bollen J., Demyttenaere K., Kasper S., Lecrubier Y., Montgomery S., Serretti A. (2007). Clinical factors associated with treatment resistance in major depressive disorder: Results from a European multicenter study. J. Clin. Psychiatry.

[4] Burrows G.D., Norman T.R., Judd F.K. (1994). Definition and differential diagnosis of treatment-resistant depression. Int. Clin. Psychopharmacol..

[5] Souery D., Amsterdam J., de Montigny C., Lecrubier Y., Montgomery S., Lipp O., Racagni G., Zohar J., Mendlewicz J. (1999). Treatment resistant depression: Methodological overview and operational criteria. Eur. Neuropsychopharmacol..

[6] Nemeroff C.B. (2007). Prevalence and management of treatment-resistant depression. J. Clin. Psychiatry.

[7] Willner P., Belzung C. (2015). Treatment-resistant depression: Are animal models of depression fit for purpose?. Psychopharmacology.

[8] Price J.L., Drevets W.C. (2012). Neural circuits underlying the pathophysiology of mood disorders. Trends Cogn. Sci..

[9] Kelly J., Wrynn A., Leonard B. (1997). The olfactory bulbectomized rat as a model of depression: An update. Pharmacol. Ther..

[10] Song C., Leonard B.E. (2005). The olfactory bulbectomised rat as a model of depression. Neurosci. Biobehav. Rev..

[11] Jacka F.N., Maes M., Pasco J.A., Williams L., Berk M. (2012). Nutrient intakes and the common mental disorders in women. J. Affect. Disord..

[12] Vashum K.P., McEvoy M., Milton A.H., McElduff P., Hure A., Byles J., Attia J. (2014). Dietary zinc is associated with a lower incidence of depression: Findings from two Australian cohorts. J. Affect. Disord..

[13] Siwek M., Szewczyk B., Dudek D., Styczeń K., Sowa-Kućma M., Młyniec K., Siwek A., Witkowski L., Pochwat B., Nowak G. (2013). Zinc as a marker of affective disorders. Pharmacol. Rep..

[14] Siwek M., Dudek D., Schlegel-Zawadzka M., Morawska A., Piekoszewski W., Opoka W., Zięba A., Pilc A., Popik P., Nowak G. (2010). Serum zinc level in depressed patients during zinc supplementation of imipramine treatment. J. Affect. Disord..

[15] Młyniec K., Davies C.L., Budziszewska B., Opoka W., Reczyński W., Sowa-Kućma M., Doboszewska U., Pilc A., Nowak G. (2012). Time course of zinc deprivation-induced alterations of mice behavior in the forced swim test. Pharmacol. Rep..

[16] Młyniec K., Nowak G. (2012). Zinc deficiency induces behavioral alterations in the tail suspension test in mice. Effect of antidepressants. Pharmacol. Rep..

[17] Doboszewska U., Szewczyk B., Sowa-Kucma M., Mlyniec K., Rafało A., Ostachowicz B., Lankosz M., Nowak G. (2015). Antidepressant activity of fluoxetine in the zinc deficiency model in rats involves the NMDA receptor complex. Behav. Brain Res..

[18] Nollet M. (2021). Models of Depression: Unpredictable Chronic Mild Stress in Mice. Curr. Protoc..

[19] Willner P., Gruca P., Lason M., Tota-Glowczyk K., Litwa E., Niemczyk M., Papp M. (2019). Validation of chronic mild stress in the Wistar-Kyoto rat as an animal model of treatment-resistant depression. Behav. Pharmacol..

[20] Eagle A., Mazei-Robison M., Robison A. (2016). Sucrose Preference Test to Measure Stress-induced Anhedonia. Bio-Protocol.

[21] Lumia A.R., Teicher M.H., Salchli F., Ayers E., Possidente B. (1992). Olfactory bulbectomy as a model for agitated hyposerotonergic depression. Brain Res..

[22] Leonard B.E., Tuite M. (1981). Leonard, Anatomical, physiological and behavioral aspects of olfactory bulbectomy in the rat. Int. Rev. Neurobiol..

[23] Pochwat B., Szewczyk B., Kotarska K., Rafało-Ulińska A., Siwiec M., Sowa J.E., Tokarski K., Siwek A., Bouron A., Friedland K. (2018). Hyperforin Potentiates Antidepressant-Like Activity of Lanicemine in Mice. Front. Mol. Neurosci..

[24] Porsolt R.D., Anton G., Blavet N., Jalfre M. (1978). Behavioural despair in rats: A new model sensitive to antidepressant treatments. Eur. J. Pharmacol..

[25] Doboszewska U., Sowa-Kućma M., Młyniec K., Pochwat B., Hołuj M., Ostachowicz B., Pilc A., Nowak G., Szewczyk B. (2015). Zinc deficiency in rats is associated with up-regulation of hippocampal NMDA receptor. Prog. Neuro-Psychopharmacol. Biol. Psychiatry.

[26] Nowak G., Szewczyk B., Sadlik K., Piekoszewski W., Trela F., Florek E., Pilc A. (2003). Reduced potency of zinc to interact with NMDA receptors in hippocampal tissue of suicide victims. Pol. J. Pharmacol..

[27] Grabrucker A.M. (2014). A role for synaptic zinc in ProSAP/Shank PSD scaffold malformation in autism spectrum disorders. Dev. Neurobiol..

[28] Hagmeyer S., Romão M.A., Cristóvão J.S., Vilella A., Zoli M., Gomes C.M., Grabrucker A.M. (2019). Distribution and Relative Abundance of S100 Proteins in the Brain of the APP23 Alzheimer’s Disease Model Mice. Front. Neurosci..

[29] Sloviter R.S. (1982). A simplified timm stain procedure compatible with formaldehyde fixation and routine paraffin embedding of rat brain. Brain Res. Bull..

[30] Rafalo A., Zadrozna M., Nowak B., Kotarska K., Wiatrowska K., Pochwat B., Sowa-Kucma M., Misztak P., Nowak G., Szewczyk B. (2017). The level of the zinc homeostasis regulating proteins in the brain of rats subjected to olfactory bulbectomy model of depression. Prog. Neuro-Psychopharmacol. Biol. Psychiatry.

[31] Pochwat B., Sowa-Kucma M., Kotarska K., Misztak P., Nowak G., Szewczyk B. (2015). Antidepressant-like activity of magnesium in the olfactory bulbectomy model is associated with the AMPA/BDNF pathway. Psychopharmacology.

[32] Tamano H., Kan F., Kawamura M., Oku N., Takeda A. (2009). Behavior in the forced swim test and neurochemical changes in the hippocampus in young rats after 2-week zinc deprivation. Neurochem. Int..

[33] Tassabehji N.M., Corniola R.S., Alshingiti A., Levenson C.W. (2008). Zinc deficiency induces depression-like symptoms in adult rats. Physiol. Behav..

[34] Whittle N., Lubec G., Singewald N. (2009). Zinc deficiency induces enhanced depression-like behaviour and altered limbic activation reversed by antidepressant treatment in mice. Amino Acids.

[35] Gorman J.M., Korotzer A., Su G. (2002). Efficacy Comparison of Escitalopram and Citalopram in the Treatment of Major Depressive Disorder: Pooled Analysis of Placebo-Controlled Trials. CNS Spectr..

[36] Lepola U.M., Loft H., Reines E.H. (2003). Escitalopram (10–20 mg/day) is effective and well tolerated in a placebo-controlled study in depression in primary care. Int. Clin. Psychopharmacol..

[37] Pandey D.K., Bhatt S., Jindal A., Gautam B. (2014). Effect of combination of ketanserin and escitalopram on behavioral anomalies after olfactory bulbectomy: Prediction of quick onset of antidepressant action. Indian J. Pharmacol..

[38] Breuer M.E., Groenink L., Oosting R.S., Westenberg H.G., Olivier B. (2007). Long-Term Behavioral Changes After Cessation of Chronic Antidepressant Treatment in Olfactory Bulbectomized Rats. Biol. Psychiatry.

[39] Poretti M.B., Sawant R.S., Rask-Andersen M., de Cuneo M.F., Schiöth H.B., Perez M.F., Carlini V.P. (2016). Reduced vasopressin receptors activation mediates the anti-depressant effects of fluoxetine and venlafaxine in bulbectomy model of depression. Psychopharmacology.

[40] McGrath C., Norman T.R. (1998). The effect of venlafaxine treatment on the behavioural and neurochemical changes in the olfactory bulbectomised rat. Psychopharmacology.

[41] Ranjbar E., Kasaei M.S., Mohammad-Shirazi M., Nasrollahzadeh J., Rashidkhani B., Shams J., Mostafavi S.-A., Mohammadi M.R. (2013). Effects of Zinc Supplementation in Patients with Major Depression: A Randomized Clinical Trial. Iran. J. Psychiatry.

[42] Szewczyk B. (2013). Zinc homeostasis and neurodegenerative disorders. Front. Aging Neurosci..

[43] Cunha M.P., Machado D.G., Bettio L.E., Capra J.C., Rodrigues A.L.S. (2008). Interaction of zinc with antidepressants in the tail suspension test. Prog. Neuro-Psychopharmacol. Biol. Psychiatry.

[44] Kroczka B., Branski P., Palucha A., Pilc A., Nowak G. (2001). Antidepressant-like properties of zinc in rodent forced swim test. Brain Res. Bull..

[45] Nowak G., Szewczyk B., Wieronska J.M., Branski P., Palucha A., Pilc A., Sadlik K., Piekoszewski W. (2003). Antidepressant-like effects of acute and chronic treatment with zinc in forced swim test and olfactory bulbectomy model in rats. Brain Res. Bull..

[46] Sowa-Kucma M., Legutko B., Szewczyk B., Novak K., Znojek P., Poleszak E., Papp M., Pilc A., Nowak G. (2008). Antidepressant-like activity of zinc: Further behavioral and molecular evidence. J. Neural Transm..

[47] Cieślik K., Klenk-Majewska B., Danilczuk Z., Wróbel A., Łupina T., Ossowska G. (2007). Influence of zinc supplementation on imipramine effect in a chronic unpredictable stress (CUS) model in rats. Pharmacol. Rep..

[48] Roozbeh J., Sharifian M., Ghanizadeh A., Sahraian A., Sagheb M.M., Shabani S., Jahromi A.H., Kashfi M., Afshariani R. (2011). Association of Zinc Deficiency and Depression in the Patients with End-stage Renal Disease on Hemodialysis. J. Ren. Nutr..

[49] Nowak G., Ziȩba A., Dudek D., Krośniak M., Szymaczek M., Schlegel-Zawadzka M. (1999). Serum trace elements in animal models and human depression. Part I. Zinc. Hum. Psychopharmacol..

[50] Nowak G., Schlegel-Zawadzka M. (1998). Alterations in Serum and Brain Trace Element Levels After Antidepressant Treatment. Biol. Trace Elem. Res..

[51] Młyniec K., Budziszewska B., Reczyński W., Doboszewska U., Pilc A., Nowak G. (2013). Zinc deficiency alters responsiveness to antidepressant drugs in mice. Pharmacol. Rep..

[52] Cieślik K., Sowa-Kućma M., Ossowska G., Legutko B., Wolak M., Opoka W., Nowak G. (2011). Chronic unpredictable stress-induced reduction in the hippocampal brain-derived neurotrophic factor (BDNF) gene expression is antagonized by zinc treatment. Pharmacol. Rep..

[53] Wróbel A., Serefko A., Wlaź P., Poleszak E. (2015). The effect of imipramine, ketamine, and zinc in the mouse model of depression. Metab. Brain Dis..

[54] Rafało-Ulińska A., Poleszak E., Szopa A., Serefko A., Rogowska M., Sowa I., Wójciak M., Muszyńska B., Krakowska A., Gdula-Argasińska J. (2020). Imipramine Influences Body Distribution of Supplemental Zinc Which May Enhance Antidepressant Action. Nutrients.

[55] Sowa-Kućma M., Kowalska M., Szlósarczyk M., Gołembiowska K., Opoka W., Baś B., Pilc A., Nowak G. (2011). Chronic treatment with zinc and antidepressants induces enhancement of presynaptic/extracellular zinc concentration in the rat prefrontal cortex. Amino Acids.

[56] Chrobak A.A., Siuda K., Tereszko A., Siwek M., Dudek D. (2014). Zaburzenia psychiczne a struktura i funkcje móżdżku—Przeģlad najnowszych bádan. Psychiatria.

[57] Leroi I., O’Hearn E., Marsh L., Lyketsos C.G., Rosenblatt A., Ross C.A., Brandt J., Margolis R.L. (2002). Psychopathology in Patients with Degenerative Cerebellar Diseases: A Comparison to Huntington’s Disease. Am. J. Psychiatry.

[58] Schutter D.J.L.G., Van Honk J. (2005). The cerebellum on the rise in human emotion. Cerebellum.

[59] Schmahmann J.D., Weilburg J.B., Sherman J.C. (2007). The neuropsychiatry of the cerebellum—Insights from the clinic. Cerebellum.

[60] Sandyk R. (1991). Zinc Deficiency and Cerebellar Disease. Int. J. Neurosci..

[61] Wang Z.-Y., Stoltenberg M., Huang L., Danscher G., Dahlström A., Shi Y., Li J.-Y. (2005). Abundant expression of zinc transporters in Bergman glia of mouse cerebellum. Brain Res. Bull..

[62] Takeda A., Nakamura M., Fujii H., Tamano H. (2013). Synaptic Zn2+ homeostasis and its significance. Metallomics.

[63] Takeda A., Minami A., Takefuta S., Tochigi M., Oku N. (2001). Zinc homeostasis in the brain of adult rats fed zinc-deficient diet. J. Neurosci. Res..

[64] Takeda A., Tamano H., Ogawa T., Takada S., Ando M., Oku N., Watanabe M. (2012). Significance of serum glucocorticoid and chelatable zinc in depression and cognition in zinc deficiency. Behav. Brain Res..

[65] Lyubartseva G., Smith J.L., Markesbery W.R., Lovell M.A. (2010). Alterations of Zinc Transporter Proteins ZnT-1, ZnT-4 and ZnT-6 in Preclinical Alzheimer’s Disease Brain. Brain Pathol..

[66] Beyer N., Coulson D.T., Heggarty S., Ravid R., Hellemans J., Irvine G.B., Johnston J.A. (2012). Zinc Transporter mRNA Levels in Alzheimer’s Disease Postmortem Brain. J. Alzheimers Dis..

[67] Bosomworth H.J., Adlard P.A., Ford D., Valentine R.A. (2013). Altered Expression of ZnT10 in Alzheimer’s Disease Brain. PLoS ONE.

[68] Whitfield D.R., Vallortigara J., Alghamdi A., Hortobágyi T., Ballard C., Thomas A.J., O’Brien J.T., Aarsland D., Francis P.T. (2015). Depression and Synaptic Zinc Regulation in Alzheimer Disease, Dementia with Lewy Bodies, and Parkinson Disease Dementia. Am. J. Geriatr. Psychiatry.

[69] Rafalo-Ulinska A., Piotrowska J., Kryczyk A., Opoka W., Sowa-Kucma M., Misztak P., Rajkowska G., Stockmeier C.A., Datka W., Nowak G. (2016). Zinc transporters protein level in postmortem brain of depressed subjects and suicide victims. J. Psychiatr. Res..

