# Supplementary material for: Zinc Deficiency Blunts the Effectiveness of Antidepressants in the Olfactory Bulbectomy Model of Depression in Rats
Source: Nutrients. 2022 Jun 30;14(13):2746. doi: 10.3390/nu14132746 (PMC9269062; doi:10.3390/nu14132746)
Supplement: Supplementary file 1 [file nutrients-14-02746-s001.zip › nutrients-1757444-supplementary.pdf]

1. The effect of antidepressants in rats subjected to the OB model on the behaviour in OFT

As shown in **Figure S1.** chronic treatment with 10 mg/kg of Esc or chronic treatment with 10 mg/kg of Ven decreased hyperactivity in rats subjected to the OB model [F (2,20) = 3,496, p= 0,0499].

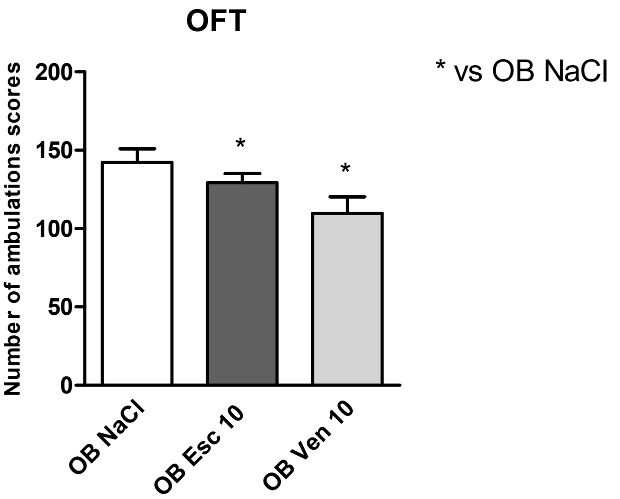

Figure S1. The effect of antidepressants (Esc, Ven) in rats subjected to the OB model in the OFT. Data was analysed using one-way ANOVA and Newman-Keuls multiple comparisons test. $*p < 0.05$ vs OB NaCl. Values are expressed as mean ± SEM (n = 7-9).

2. The effect of antidepressants in rats subjected to the OB model on the behaviour in SIT

As shown in **Figure S2.** chronic treatment with 10 mg/kg of Esc or chronic treatment with 10 mg/kg of Ven increased the sucrose intake in rats subjected to the OB model [F (2,15) = 4,841, p= 0,0239].

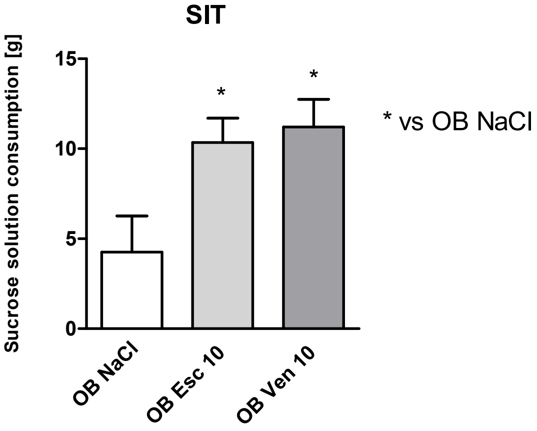

Figure S2. The effect of antidepressants (Esc, Ven) in rats subjected to the OB model in the SIT. Data was analysed using one-way ANOVA and Newman-Keuls multiple comparisons test. $*p < 0.05$ vs OB NaCl. Values are expressed as mean ± SEM (n = 7-9).
